# “Tau Oligomers,” What We Know and What We Don’t Know

**DOI:** 10.3389/fneur.2014.00001

**Published:** 2014-01-13

**Authors:** Naruhiko Sahara, Jesus Avila

**Affiliations:** ^1^Molecular Imaging Center, National Institute of Radiological Sciences, Chiba, Japan; ^2^Centro de Biologia Molecular Severo Ochoa (CSIC-UAM), Madrid, Spain

**Keywords:** tau protein, tauopathy, neurodegenerative disease, propagation, tau phosphorylation

**Figure 1:**
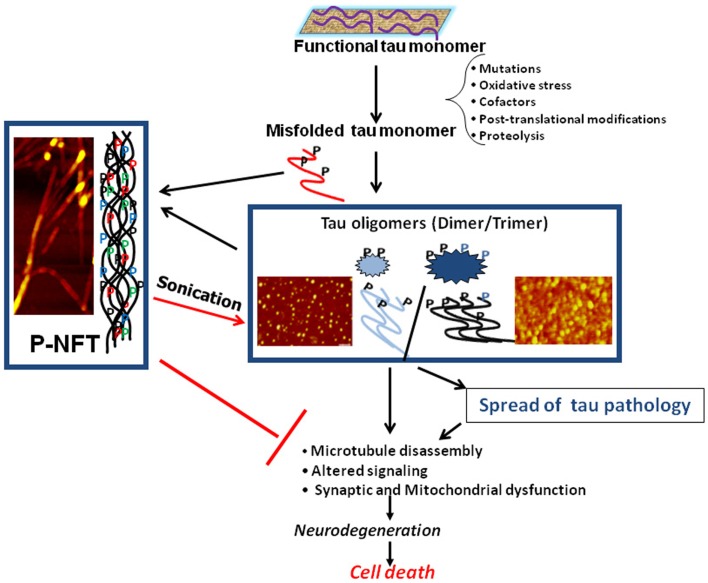
**Schematic illustration of the central role of tau oligomers in tauopathies**. Figure taken from Gerson and Kayed (1).

Neurofibrillary tangles, composed of intracellular aggregates of tau protein, are a key neuropathological feature of Alzheimer’s disease and other neurodegenerative diseases, collectively termed tauopathies. Tau research has become one of the central players in the investigation of neurodegenerative diseases. Tau protein has several unique characteristics such as natively unfolded conformation, thermo-stability, acid-stability, and capability of post-translational modifications. We still do not know whether tau itself is toxic. With certain triggers, tau may transit into toxic forms. Researchers are now looking for “tau oligomers” as toxic components. Because “tau oligomers” contain variable species of tau protein [e.g., dimer (disulfide bond-dependent or -independent), multimer (more than dimer), granular (defined as EM or AFM) and perhaps small filamentous aggregates] (Figure 1), it is important to have a consensus regarding the definition, terminology, and methodology for the identification of “tau oligomers” (1–6).

Recently, “prion-like” toxicity and propagation mechanisms underlying the progression of disease have been proposed. With this concept, tau may have the ability to translocate between neurons and amplify toxic components (7). Although we do not know the exact forms of toxic tau oligomers, accumulating evidence has shown the probability of tau oligomer propagation (6).

Tau is an intracellular microtubule-associated protein. The mechanism of tau transmission from cell to cell is still unknown. Research focusing on extracellular tau will open potential new avenues for discovering the mechanism of tau propagation (8).

Abnormally hyperphosphorylated tau is a key feature of human tauopathies. Although we are not sure whether phosphorylation rather than oligomerization of tau is an initial molecular event in tau pathogenesis, investigating the regulatory mechanisms of tau phosphorylation will be essential (9–11).

Here, we provide an overview of the current understandings of “tau oligomers” (1–12). Efforts toward the identification of neurotoxic tau species will ultimately lead to the translational research for developing novel therapeutic strategies for tauopathies.
